# A Study of PLA Thin Film on SS 316L Coronary Stents Using a Dip Coating Technique

**DOI:** 10.3390/polym16020284

**Published:** 2024-01-19

**Authors:** Mariana Macías-Naranjo, Margarita Sánchez-Domínguez, J. F. Rubio-Valle, Ciro A. Rodríguez, J. E. Martín-Alfonso, Erika García-López, Elisa Vazquez-Lepe

**Affiliations:** 1 Tecnologico de Monterrey, School of Engineering and Sciences, Ave. Eugenio Garza Sada 2501, Monterrey 64849, Nuevo León, Mexico; a01280232@tec.mx (M.M.-N.); ciro.rodriguez@tec.mx (C.A.R.); 2 Centro de Investigación en Materiales Avanzados, S.C. (CIMAV), Unidad Monterrey, Alianza Norte 202, Apodaca 66628, Nuevo León, Mexico; margarita.sanchez@cimav.edu.mx; 3 Pro2TecS—Chemical Product and Process Technology Research Center, Department of Chemical Engineering and Materials Science, ETSI, Universidad de Huelva, Campus de “El Carmen”, 21071 Huelva, Spain; josefernando.rubio@diq.uhu.es (J.F.R.-V.); jose.martin@diq.uhu.es (J.E.M.-A.)

**Keywords:** coronary artery stent, dip coating, polylactic acid, thin film, polymeric coating

## Abstract

The dip coating process is one of the recognized techniques used to generate polymeric coatings on stents in an easy and low-cost way. However, there is a lack of information about the influence of the process parameters of this technique on complex geometries such as stents. This paper studies the dip coating process parameters used to provide a uniform coating of PLA with a 4–10 µm thickness. A stainless-steel tube (AISI 316L) was laser-cut, electropolished, and dip-coated in a polylactic acid (PLA) solution whilst changing the process parameters. The samples were characterized to examine the coating’s uniformity, thickness, surface roughness, weight, and chemical composition. FTIR and Raman investigations indicated the presence of PLA on the stent’s surface, the chemical stability of PLA during the coating process, and the absence of residual chloroform in the coatings. Additionally, the water contact angle was measured to determine the hydrophilicity of the coating. Our results indicate that, when using entry and withdrawal speeds of 500 mm min^−1^ and a 15 s immersion time, a uniform coating thickness was achieved throughout the tube and in the stent with an average thickness of 7.8 µm.

## 1. Introduction

Approximately 5 million percutaneous coronary interventions (PCI) are performed annually worldwide [1,2,3]. Atherosclerosis is one of the most recurring inflammatory problems produced in the coronary arteries in the last years around the world [4,5,6,7]. Stenting is an important treatment to allow blood to flow through narrowed coronary arteries [2,8]. Stents are classified into four categories: bare metallic stents (BMS), coated metallic stents, biodegradable stents (BDS), and drug-eluting stents (DES) [5,8]. BMS placement and DES have excelled in combating coronary artery disease compared with BDS and coated metallic stents [9,10].

The first generations of bare-metal stents were manufactured with alloys such as 316L stainless steel (316L SS), cobalt–chromium (Co-Cr), platinum–iridium (Pt-Ir), tantalum (Ta), nitinol (Ni-Ti), magnesium (Mg), and pure iron (Fe) [1,8,11]. However, they have presented problems such as thrombosis, tissue hyperplasia, neointimal proliferation, in-stent restenosis, and the need for removal due to rejection by the body [5,11,12,13]. Around 30–60% of cases are estimated to suffer from in-stent restenosis after a BMS has been implanted [3,8,9,12]. While the use of a DES decreases this condition, late stent thrombosis (LST) can still occur [8,9].

Stainless steel is still one of the most common metals used in stent manufacturing due to its excellent mechanical properties, corrosion resistance [8,14], ferromagnetic nature, and low-density influence on biocompatibility. However, its non-MRI compatibility and poor visibility in fluoroscopic images do not allow its in vivo placement [8]. Therefore, to improve the biocompatibility and hemocompatibility properties of stainless steel, coatings are commonly used to modify its surface features, reduce the surface free energy, smoothen the texture, neutralize the surface potential, and improve surface stability [8,15]. Furthermore, drugs can be added, and these modifications reduce thrombosis and neointimal proliferation, which reduces restenosis [16,17], and drug release controls intimal hyperplasia.

The coating materials used to improve the functioning of stents are classified into inorganic materials, polymers, porous metals, and endothelial cells [8]. Polymer coatings improve the properties of stents because they act as a vehicle for drug release and can work with different biomolecules [18]. A wide variety of polymers have been used for stent coating materials, such as polylactides (PLAs), poly(ethylene) (PE), polyurethanes (PURs), and poly(glycolide) (PGA), among others [3,5,12]. Among the polyesters, polylactic acid (PLA) is a polymer with attractive properties such as biodegradability, bioabsorbability, and non-toxicity [19,20]. Additionally, it has shown excellent mechanical properties compared to other synthetic polymers at a more affordable price [20,21]. It is essential to select an adequate material for use on stents because there are many requirements that must be met, including that the material should be non-cytotoxic, resorbable, flexible, radiopaque, biocompatible, non-erodible, and it should display excellent hemocompatibility, controlled drug encapsulation and release, and sufficient radial strength [5,8,21,22].

Dip coating is one of the most used techniques because it is simple and affordable due to the low amount of material used and the simplicity of the required equipment [23]. Additionally, this process is one of the simplest coating processes, offering surface uniformity [24]. This technique consists of three stages: immersion and dwelling, drainage, and drying. The substrate is immersed in the solution at a certain speed during the immersion and dwelling stages. Then, it remains there for a period, allowing the materials to interact and the coating of the substrate to be achieved. Subsequently, the substrate is withdrawn at a certain speed, releasing the excess solution. Finally, solvent evaporation occurs in the substrate’s drying stage, allowing the adhesion of the coating to be completed and eliminating all residues [25]. The process parameters involved are the immersion time, entry speed, withdrawal speed, and the number of immersion cycles [23]. Dip coating stands out, having been highlighted as one of the simplest, quickest, most affordable, and highest-quality coating techniques, and it is used both on an industrial scale and at the research-laboratory level. It is one of the most common methods used to produce thin films from a wide range of inorganic, hybrid, and nanocomposite materials [26]. Compared to other techniques that are limited by the geometry or size of the sample, this technique covers a wide range of substrates and complex geometries, including holes and intricate patterns. The dip coating method provides a high degree of control over its parameters and offers a flexibility that is impossible with other traditional processes. For example, this technique can control the coating thickness by controlling the solution’s withdrawal speed and viscosity [26]. The coating’s properties, such as the thickness, uniformity, porosity, and roughness, depend on the solution’s properties [26,27,28]. These properties (e.g., shear viscosity, surface tension), combined with gravity, are important forces involved in this process, and they change as the solution’s concentration is varied. It is crucial to control and analyze the effects of this parameter since it has the most significant influence on the obtained film when the dip coating technique is used [28]. The formation of a smooth film depends on the correct spreading of the film along the substrate, where the concentration of the solution increases as the solvent evaporates [27].

Table 1 summarizes several studies on polymeric coatings designed for stent purposes using dip coating and the process parameters related to the substrate. Among these studies, Núñez et al. [24] used a dip coating machine and 10% PCL TiO_2_ 1% as the coating. They set the entry and withdrawal speeds at 125 mm min^−1^, the immersion time to 5 s, and completed ten immersion cycles. The contribution of this study was the analysis of the etching and coating of a Palmaz–Schatz stent manufactured by laser cutting, analyzing surface roughness, thickness, and degradation.

Guerra et al. [29] analyzed the effect of the dip coating process’ parameters on the tube features of stents using PCL and PLA as the coating. After coating, they characterized the tubes to demonstrate the influence of different parameters and materials to approach the stent’s requirements. They designed experiments to analyze different levels of withdrawal speeds, polymer concentrations, and the number of cycles carried out. They found a strong influence for the thickness of the coating and surface roughness on the withdrawal speed and the polymer’s concentration. Mojarad et al. [30] coated 316 L substrates with PCL-gelatin using the dip coating technique. They recognized that the dip coating technique is excellent for obtaining a smooth and uniform coating. Comparing the different works presented in the literature review in Table 1, it is clear that the study of coating morphology and film characterization is limited to the dip coating technique for complex geometries such as stents. To the authors’ knowledge, there are no studies involving the characterization of thin films on stainless steel stents coated using PLA at different concentrations.

**Table 1:** Literature Review of polymeric coating for stent purposes using dip coating technique.

Reference	Substrate	Coating	Concentration	Process Parameters	Coating Properties
[24]	AZ31 Coronary stents (L = 10 mm, D = 3, 1.8 mm)	PCL with 1% of TiO_2_	10%	Entry speed: 125 mm min^−1^ Withdrawal speed: 125 mm min^−1^ Immersion Time: 5 s Cycles: 10	Morphology Surface roughness (~1.5 μm R_a_ and ~10 μm R_z_) Thickness (20–110 μm) Degradation
[29]	PCL-PLA Tube (D = 4 mm)	PLA and PCL	3%, 5%, 7% (*w*/*v*)	Withdrawal speed: 50, 250, 450 mm min^−1^ Cycles: 40, 50, 60	Morphology Surface Roughness Thickness (100 μm)
[31]	Nitinol Stents (L = 60 mm, D = 30 mm)	DTX and PU	DTX/PU loadings 1.92% and 2.79% (*w*/*w*)	Withdrawal speed: 390 mm min^−1^ Immersion Time: 60 s Cycles: 2	Morphology Weight (559–563.93 mg/stent) In vitro release Cytotoxicity Cell behavior
[30]	316L SS Square specimens (10 × 10 × 2 mm)	PCL-Gelatin composite	50%, 70%, 90% (*w*)	Entry speed: 120 mm min^−1^ Withdrawal speed: 300 mm min^−1^ Immersion Time: 120 s Cycles: 5	Morphology Surface roughness (4.778–6.518 μm) Coating attachment Chemical composition Electrochemical behavior
[32]	Zinc Wires	PLLA	1.5, 5, and 15 g in 100 mL		Morphology (SEM pores of 12 μm) Thickness (1–12 μm) Electrochemical behavior Cytotoxicity Biocorrosion Biocompatibility
[33]	SS stents	Fluorinated polyphosphazenes and polymethacrylates	0.75% (*w*/*v*)		Morphology Stent implantation Quantitative coronary angiography

The present work investigates the influence of the process’ parameters in the dip coating technique using AISI 316L stainless steel as a substrate and PLA as the coating. The study’s objective was to obtain a uniform coating with a thickness of 4–10 µm, which is a manufacturing constraint for polymer coating in coronary stents [25,34,35].

## 2. Materials and Methods

### 2.1. Materials

The experiments used AISI 316L stainless steel tubes (AISI 316L SS) with a 3.175 mm outer diameter and a 0.254 mm wall thickness (McMaster-Carr, Robbinsville, NJ, USA). For coating, PLA in pellet form (Ingeo™ Biopolymer 3251D) was supplied by Nature Works (Plymouth, MN, USA). Chloroform (Chloroform RA, CHCl_3_ 67-66-3) was purchased from CTR Scientific (Monterrey, NL, Mexico) and was used to prepare PLA solutions of different concentrations.

### 2.2. Fabrication of Samples

**Figure 1:**
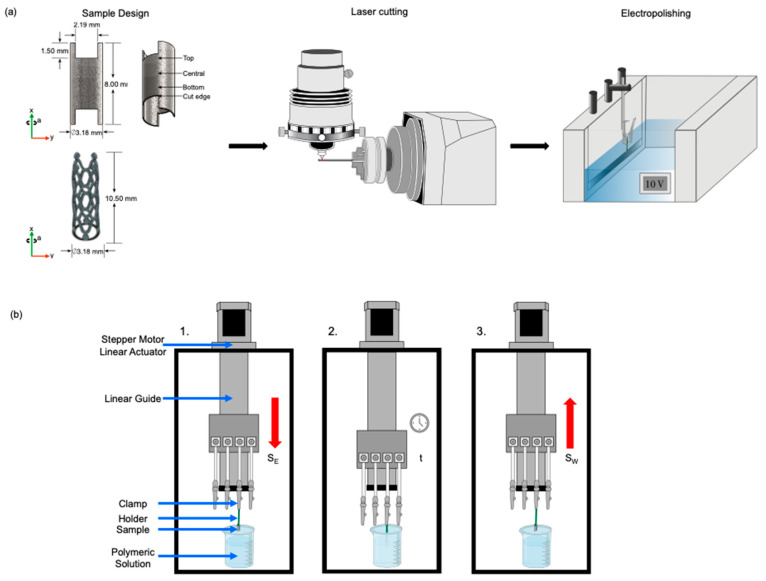
Process and samples: (**a**) Process chain of samples and (**b**) dip coating system: entry, immersion, and withdrawal stages.

Figure 1a represents a diagram that shows the stages of preparing the samples. Samples were designed in Solidworks to be cut from a AISI 316L SS tube. A 4-axis laser cutting machine (Preco, MedPro ST2000, St. Croix, WI, USA) was used to laser-cut the sample geometries. To assure surface quality after laser cutting, a mild electrolyte for electropolishing stainless steel (ESMA, SS Electropolish E972, Chicago, IL, USA) was used at a temperature of 60 °C, with 10 V, for a time of 50 s. Subsequently, the samples were immersed in a 20% nitric acid (HNO_3_) solution for 10 s and dipped in a saturated sodium bicarbonate (NaHCO_3_) solution for 10 s. The samples were then washed in an ultrasonic bath for 2 min using isopropyl alcohol and distilled water for 2 min. The same electropolishing conditions were applied to the stent’s geometry, requiring an etching time of 90 s. The cleaning process was carried out in the same way as the previous geometry. This treatment was performed to remove slag and dross particles caused by metal fusion and reduce the cutting edge’s surface roughness.

### 2.3. PLA Solutions Study

The influence of PLA concentration on the dip coating process was investigated using chloroform (CHCl_3_) as solvent. Before using the PLA, the pellets were dried in an oven at 80 °C for 24 h. PLA was dissolved at 25 °C, under magnetic stirring (300 RPM), to obtain solutions with concentrations ranging from 0.5% to 15% (*w*/*v*) (0.5%, 1%, 3%, 5%, 7.5%, 10%, 12.5%, and 15% (*w*/*v*)).

Dynamic shear viscosity measurements of PLA solutions were carried out using an AR G2 (TA Instruments, New Castle, DE, USA) controlled-strain rheometer with a shear rate range of 1–400 s^−1^ using Couette geometry (stator inner radius 15 mm, rotor outer radius 14 mm, cylinder immersed height 42.05 mm) at 25 °C. Surface tension measurements were performed using the pendant drop method (OCA 15 Plus by DataPhysics, CA, USA) at 25 °C. At least three replicates were performed.

### 2.4. Dip Coating Process

Figure 1b shows the dip coating machine built for previous works [24]. The machine was programmed using an Arduino card to set the entry and withdrawal speeds. The samples were placed in a vertical position and were immersed using a linear guide. The entry speed, S_E_ [mm min^−1^], withdrawal speed, S_W_ [mm min^−1^], time, t [s], and the number of cycles, C, were varied in the dip coating process. In the entry stage, the sample is lowered into the solution at a constant speed, indicated in Figure 1b by a red arrow. Then, the sample remains immersed in the solution without movement for the set time. In the third stage of the process, the piece is withdrawn at a set constant speed, indicated in Figure 1b by a red arrow. Before coating, the samples were ultrasonically cleaned (Branson, 2800, Brookfield, CT, USA) with acetone for 5 min and ethanol for 5 min. Samples were entirely dip coated in PLA in a vertical position with different process parameters. After experimentation, samples were air-dried.

A process window was carried out on the geometry presented in Figure 1a. Table 2 shows the dip coating ranges used in the process window. After this, trials were selected to achieve a thickness between 4–10 µm in the same geometry [35,36,37,38,39]. Finally, three coronary stents were coated using the best parameters selected during the previous stage to validate the parameters chosen in a more complex geometry.

### 2.5. Characterization of Dip-Coated Samples

The coating uniformity was studied from the process window according to the coating applied. Tubes were analyzed with a stereo microscope (Olympus, SZH, TYO, Japan). The samples were analyzed in three sections (top, center, and bottom zones (see Figure 1a) to observe the material’s deposition throughout the sample. The samples were weighed using an analytical balance (Mettler Toledo, XPR205, Greifensee, ZH, Switzerland) to analyze the increase in weight due to the adhering material. Tube samples and stent images obtained with a scanning electron microscope (SEM) (Zeiss, EVOMA25, Jena, Germany) were analyzed for the selected parameters to observe surface uniformity. Samples and stents were covered with a thin layer of gold (1 nm) by sputtering to improve the low contrast due to the polymeric thin film deposited on geometries. The thickness of the tubes and stents was measured using SEM images of the parameters selection and stent coating using ImageJ software (National Institutes of Health, ImageJ 1.53t, Bethesda, MD, USA). Three measurements were taken for each test to calculate the average thickness. Average surface roughness, R_a_, and ten-point mean roughness, R_z_, were measured using a confocal scanning microscope (Zeiss, Axio CSM 700, Jena, Germany) with a magnification of 20× and a resolution of 0.05 µm. For these measurements, three values from each test were used to obtain more accurate results. Morphological analysis was carried out with field emission scanning electron microscopy (FESEM) (FEI Company, Nova NanoSEM 200, Hillsboro, OR, USA) to study the influence of different concentrations on the sample’s porosity. Stents were covered with a thin layer of gold by sputtering (1 nm).

### 2.6. Thin Film Characterization

The surface analysis of the PLA coating at different concentrations was carried out using an atomic force microscope (AFM) model MFP3D-SA (ASYLUM RESEARCH, Santa Barbara, CA, USA), performing three measurements for each concentration deposited on SS plates. The chemical structure of coated stents was studied using Fourier transform infrared spectroscopy (FT-IR) with a Perking Elmer FT-IR/FIR spectrometer model Spectrum 400 (Perkin Elmer, Hopkinton, MA, USA). Each sample was scanned 16 times between 4000–400 cm^−1^, with a 4 cm^−1^ resolution in room-temperature conditions (21 °C). The presence of residual solvent was studied with Raman spectroscopy using a LabRAM HR Evolution spectroscopic microscope (Horiba, Irvine, CA, USA) using a 50× longer working distance objective, with a 633 nm laser at 5% of power (100% = 17 mW), and an integration time of 15 s to compare and analyze the pellets and films deposited on SS plates. Each sample was scanned between 100 and 3500 cm^−1^. Water contact angle measurements were carried out with a deionized water droplet (10 μL) using the sessile drop method (OCA 15 Plus by DataPhysics, Riverside, CA, USA). An average of 10 measurements were reported for three concentrations. The droplet images were captured on camera and processed to measure the contact angle value.

## 3. Results and Discussion

### 3.1. PLA Solutions Study

**Figure 2:**
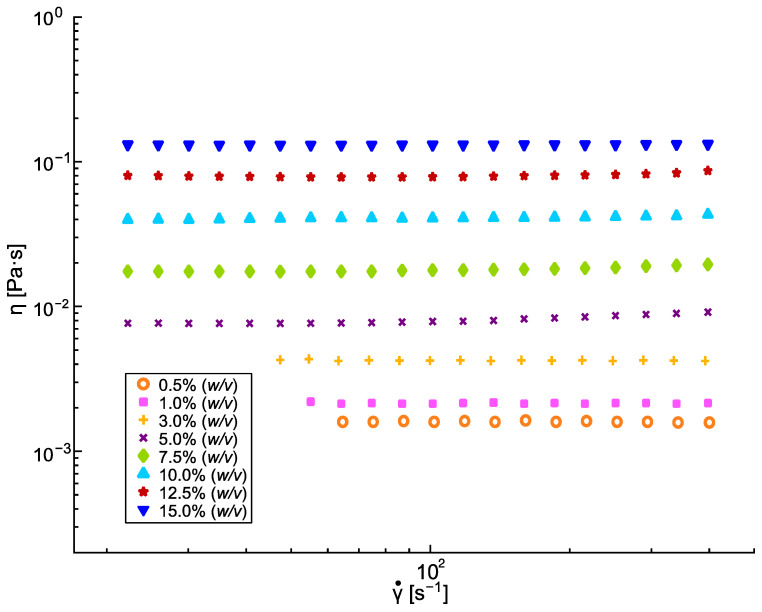
Shear viscosity of PLA solutions in CHCl_3_.

It is well-known that the performance of the dip coating process depends on the processing conditions (entry speed, withdrawal speed, immersion time, number of cycles) and the critical physicochemical properties of the solution, such as solvent boiling temperature and vapor pressure, viscosity, density, and surface tension [40,41,42,43]. In particular, the solution’s viscosity is a critical parameter, which has often been adapted by modifying the polymer’s concentration [44,45], and some correlations were applied to find the minimum polymer concentration needed to reach the viscosity values that are suitable for the dip coating process. For example, Zhang et al. [46] determined that a specific viscosity threshold corresponds to obtaining homogeneous coatings with the lowest number of defects. Figure 2 shows the shear viscosity values for the different PLA solutions at different concentrations. All of the PLA solutions studied demonstrated Newtonian behavior in the applied shear rate range (1–400 s^−1^). As expected, the higher the PLA concentration, the higher the viscosity value. Moreover, these results agree with those reported by other authors [47].

**Figure 3:**
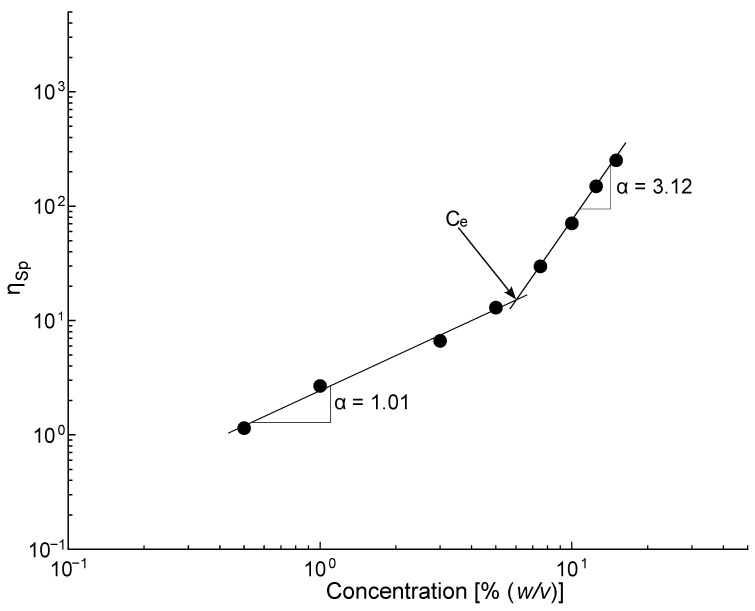
Plot of the Specific viscosity of PLA concentration and estimation of the critical entanglement concentration.

Figure 3 shows the relationship between specific viscosity (η_sp_) and PLA concentration. The critical entanglement concentration (C_e_) delimiting the semi-diluted unentangled and the semi-diluted entangled regimes can be obtained from the change in the slope of this plot. As can be seen, the C_e_ is around 7% (*w*/*v*), from which an increase in the scaling exponent is obtained, from η_sp_ α C1.01 to a η_sp_ α C3.12, which are values quite in line with those expected for a neutral polymer in a suitable solvent [48,49]. Thus, a concentration near the C_e_ (7.5% (*w*/*v*)) was selected to be applied in the dip coating technique process; this concentration has not been studied for coatings on coronary stents.

Surface tension values for PLA solutions at different concentrations are provided in Table S1. Surface tension is one of the factors that can affect the structure of the depositing film [50]. The results showed that the surface tension in the PLA solutions did not significantly change in the studied concentration range (24.56–26.17 mN m^−1^). At 7.5% (*w*/*v*) of PLA, the lowest surface tension value was obtained (24.56 mN m^−1^), which allows for better adhesion to the surface. At a higher surface tension, it can be difficult to coat the substrate homogeneously [51].

### 3.2. Dip Coating Parameters Study

#### 3.2.1. Process Window

**Figure 4:**
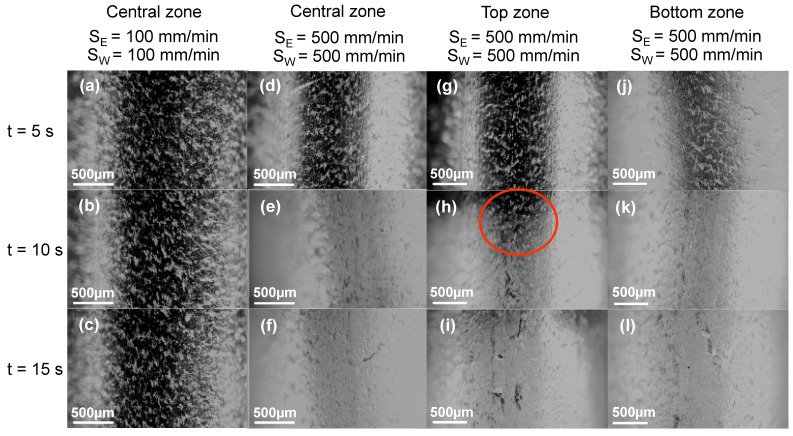
Coating uniformity observed in the stereo microscope at different entry and withdrawal speeds and immersion times: (**a**–**c**) Central zone, S_E_ = 100 mm min^−1^ and S_W_ = 100 mm min^−1^, for 5 s, 10 s, and 15 s, respectively, (**d**–**f**) Central zone, S_E_ = 500 mm min^−1^ and S_W_ = 500 mm min^−1^, for 5 s, 10 s, and 15 s, respectively, (**g**–**i**) Top zone, S_E_ = 500 mm min^−1^ and S_W_ = 500 mm min^−1^, for 5 s, 10 s, and 15 s, respectively, and (**j**–**l**) Bottom zone, S_E_= 500 mm min^−1^ and S_W_ = 500 mm min^−1^, for 5 s, 10 s, and 15 s, respectively.

Figure 4a–f presents the coating’s uniformity after dip coating using different entry and withdrawal speeds in the central zone. The images in the process window showed that, the longer the immersion time, the thicker the coating. This could be explained by the material adhering better to the tube if it stopped creating turbulence in the solution. The tonality of the images varied due to the thickness of the coating. Identical results were obtained in previous studies [52]. The appearance of the coating is related to the thickness, which increases as the withdrawal speed increases. Figure 4c,f shows the results obtained with an immersion time of 15 s in the central zone. As the withdrawal speed increases, the thickness of the coating also increases. It is important to emphasize that a faster withdrawal speed results in a thicker coating adhering to the surface. According to Kim et al. (2019) [52], the withdrawal speed influences the film’s uniformity and thickness, which is greater when the speed increases. This is due to the impact of solvent vaporization when the substrate is removed from the solution [52].

Comparing Figure 4a,d, by maintaining the immersion time, there is a qualitative difference in the coating with the higher entry and withdrawal speed. Samples with a S_E_ = 500 mm min^−1^ and S_W_ = 500 mm min^−1^ resulted in a more uniform surface (i.e., Figure 4d–f). Due to the consistent results in Figure 4a–f, the top and bottom zones of the tube were analyzed at different immersion times (Figure 4g–l). This comparison was made to evaluate the deposited material qualitatively. Our results indicate that the coated material was homogeneous in the center zone compared to that in the vertical immersion, and the viscosity effect and gravity caused a dropping impact. In Figure 4h, the red circle shows how the coating slipped down in a degraded manner due to this effect. The sample with the most uniformity had the highest immersion time (i.e., 15 s) (Figure 4f,i,l).

**Figure 5:**
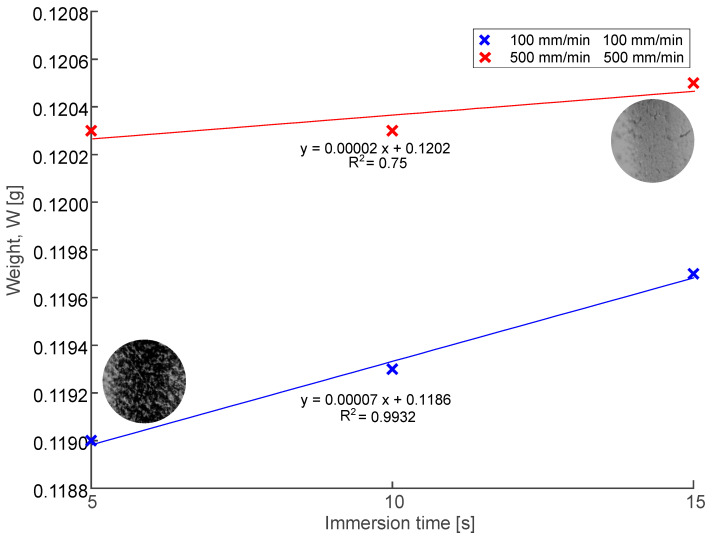
Weight comparison of the samples.

Figure 5 shows the results for the average weight of the samples according to the parameters used. Regarding the immersion time, it was found that the longer the immersion time, the greater the material’s adherence to the tube. As for the entry and withdrawal speeds, the weight of samples increases as the speeds individually increase. Additionally, when immersion time is increased with the lowest entry and withdrawal speeds (100 mm min^−1^, 100 mm min^−1^), the solution stabilizes and the weight increases. When the withdrawal speed is faster, more material adheres to the tube’s surface, preventing the solution from remaining attached to it. Preliminary test results show a coefficient of determination between 75% and 99%. Strobel et al. [53], in their work, mentioned that time has almost no effect on the coating mass and thickness. However, withdrawal speed significantly influenced the coating’s mass and thickness.

#### 3.2.2. Parameters Selected

**Figure 6:**
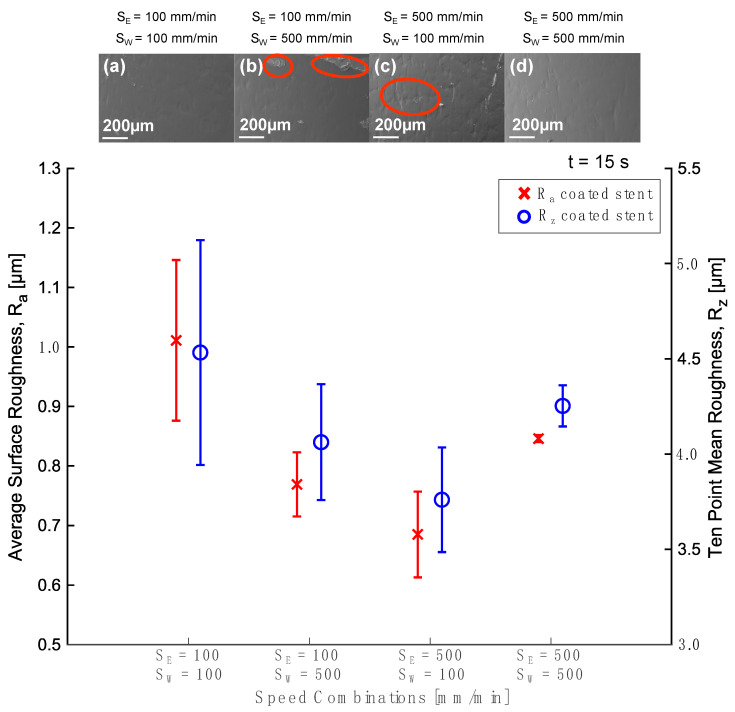
Average surface roughness (R_a_) and ten-point mean roughness (R_z_) after coating and PLA coating showed at different entry and withdrawal speeds: (**a**) S_E_ = 100 mm min^−1^ and S_W_ = 100 mm min^−1^, (**b**) S_E_ = 100 mm min^−1^ and S_W_ = 500 mm min^−1^, (**c**) S_E_ = 500 mm min^−1^ and S_W_ = 100 mm min^−1^, and (**d**) S_E_ = 500 mm min^−1^ and S_W_ = 500 mm min^−1^.

To observe the coating’s uniformity, a magnification of 15× was used to monitor the complete sample. The SEM images show a homogeneous coating without cracks. It was found that the coating is thinner (7.039 mm) using slower speeds for either entry or withdrawal (Figure 6a). Compared to previous results, the coating is thicker and more uniform when utilizing the combination of high entry and withdrawal speeds of 500 mm min^−1^ (Figure 6d). Figure 6b,c shows the irregularities in the process indicated by red circles. A coating lamination (Figure 6b) can be observed due to handling of the sample where the coating was damaged. In addition, there are porosities due to the surface of the tube (Figure 6c). This is because the effect of gravity is manifested in the dip coating process since the drag force and gravity determine the amount of PLA that remains in the sample [53].

Surface roughness is an essential property in coatings for medical devices because it affects blood and tissue compatibility, and it could influence thrombus formation and neointimal hyperplasia [33]. Before electropolishing, the samples had an average surface roughness (R_a_) of 1.101 µm. When the samples were electropolished, this value changed to 0.837 µm. Figure 6 illustrates that the surface roughness decreased with an entry speed of 500 mm min^−1^ and a withdrawal speed of 100 mm min^−1^, obtaining an R_a_ of 0.685 µm and an R_z_ of 3.760 µm. However, when both entry and withdrawal speeds of 500 mm min^−1^ were used, there was less variability in the measurements. According to Motealleh et al. (2016), solvent evaporation is an unavoidable phenomenon that influences the concentration of the solution [54], causing surface irregularities such as roughness on the samples. Coating adhesion is also essential because the interaction between the substrate and the polymer determines the coating’s functionality. This property depends on the composition of the surface as well as its substrate morphology. For example, the substrate must be clean, and its roughness must be minimal compared to the thickness of the coating for good coating adhesion [55,56]. Voicu et al. (2023) analyzed the adhesion of the PLA coating on the AZ31 substrate by comparing coatings deposited by dip coating and electrospinning, concluding that dip coating favors coating adhesion compared to electrospinning [57]. Further studies must be performed to evaluate coating adhesion and to prove the importance of minimal surface roughness.

**Figure 7:**
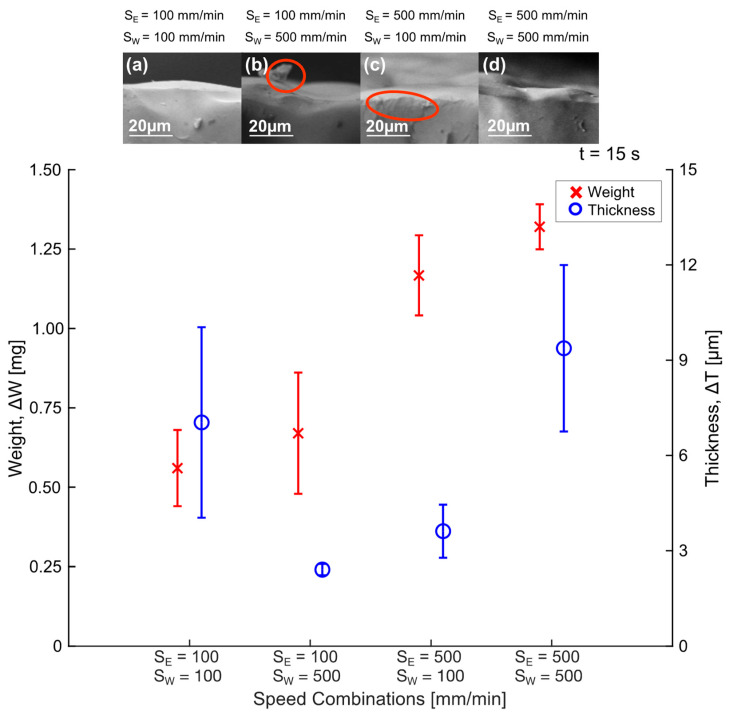
Weight and thickness increases throughout the different speed combinations (t = 15 s) and PLA coating showed by SEM images at different entry and withdrawal speeds: (**a**) S_E_ = 100 mm min^−1^ and S_W_ = 100 mm min^−1^, (**b**) S_E_ = 100 mm min^−1^ and S_W_ = 500 mm min^−1^, (**c**) S_E_ = 500 mm min^−1^ and S_W_ = 100 mm min^−1^, and (**d**) S_E_ = 500 mm min^−1^ and S_W_ = 500 mm min^−1^.

Figure 7 illustrates the increase in weight and thickness of the tube once the coating has been applied. When the speed increased, the thickness increased from 3.6 µm to 9.3 µm with both speeds at 100 mm min^−1^ and 500 mm min^−1^, respectively. A similar effect has been demonstrated in previous works [41,52,58]. Fang et al. (2008) mentioned that the thickness increases as the withdrawal speed increases since more liquid is extracted. Therefore, it results in a thicker film [41]. Likewise, Guerra et al. (2017) concluded that the layer thickness depends on the withdrawal speed and solution concentration [29]. Additionally, the SEM images (Figure 7a,d) illustrate the coating surface, showing major uniformity. Figure 7b,c shows the lamination and cracks indicated by red circles. Strobel et al. (2011) found that the withdrawal speed strongly influenced coating mass and thickness, but their results demonstrated the opposite effect. When they used 600 mm min^−1^ as the withdrawal speed, they obtained a 4.3 ± 0.8 μm thickness, and when they used 1200 mm min^−1^ as the withdrawal speed, the thickness was reduced to 3.2 ± 2.1 μm [53]. Our experiments do not evaluate this range of speeds, but we do have an opposite trend for lower speeds. We consider that, if the entry speed is modified, turbulence can be generated in the fluid. The desired thickness in coating stents was achieved using both speeds at 100 mm min^−1^ or 500 mm min^−1^ (a thickness range between 4–10 µm).

Additionally, with both speeds at 100 mm min^−1^, there was an increase in weight corresponding to 0.56 mg, while at the highest speeds (500 mm min^−1^), the weight increased to 1.32 mg. Therefore, the higher the entry and withdrawal speed (maintaining the immersion time), the greater the thickness and weight obtained. This can be explained because a faster movement allows chloroform to evaporate from the PLA solution. When the entry speed differs from the withdrawal speed, a thinner thickness results compared to when both speeds are equal; this difference is due to the PLA solution accumulating on one side of the tube. It is essential to mention that, in the samples with the speed combination of 100 and 500 mm min^−1^ and 500 and 100 mm min^−1^, the thickness decreased even as the weight increased. This is because the thickness may be thinner in the top zone.

#### 3.2.3. Stents Coated

**Figure 8:**
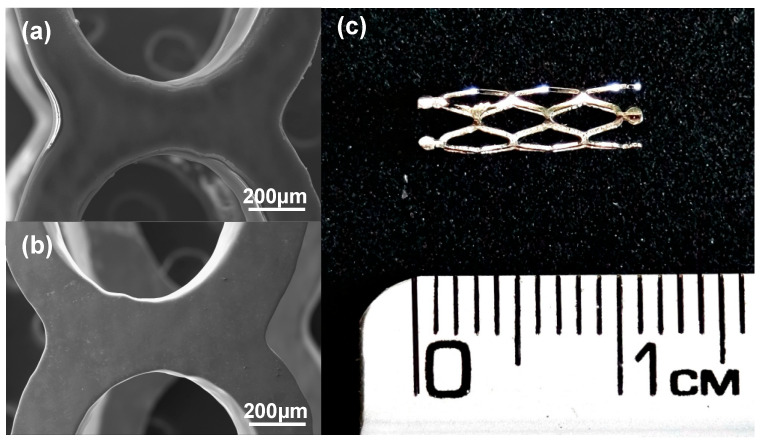
Stent geometry: (**a**) uncoated stent observed in SEM, (**b**) coated stent observed in SEM (Magnification 100×), and (**c**) photograph of coated stent.

Stents were coated using both speeds at 500 mm min^−1^ for 15 s. Characterization by SEM (Figure 8) shows uniformity in the coating. Some laminations on the edges of the coating were found due to bubble formation between the stent struts which were created during the polymer film deposition process. After the coating process, surface roughness was between 0.627 and 0.895 µm. Nuñez et al. (2021) reported that coated stents showed variations in roughness due to small porosities formed during the dip coating process [24]. A 2.195% increase in weight was obtained after coating, and the average thickness obtained was 7.862 µm. These results demonstrated that the selected parameters accomplished the objective thickness, and therefore, this technique is applicable to cover more complex geometries, such as a stent.

**Figure 9:**
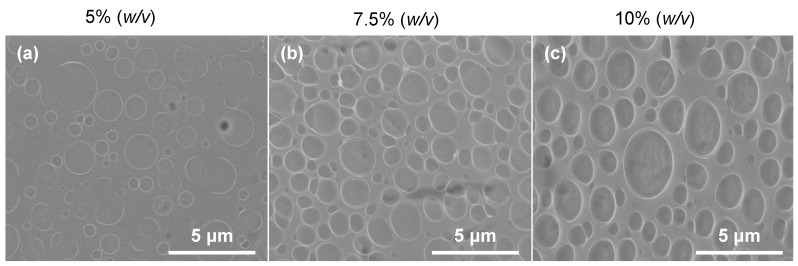
SEM micrographs: (**a**) 5% (*w*/*v*), (**b**) 7.5% (*w*/*v*), and (**c**) 10% (*w*/*v*).

Figure 9 shows the micrographs for the PLA film on stents for the three concentrations: 5%, 7.5%, and 10% (*w*/*v*). As seen in all the micrographs, there is porosity throughout the stents due to solvent evaporation. The observed porosity depends on the mechanism of solvent evaporation during the drying process [59]. Fouladian et al. (2021) mentioned that cavities appear during solvent evaporation due to water condensation as the surface cools, leading to respiration and the formation of defined structures on the surface [31]. The increase in pore depth can be correlated with sample viscosity as PLA concentration increases. In a more diluted solution, trapped air is released more efficiently, and the pore depth is smaller than in one with a higher concentration. In all concentrations, the pores are distributed heterogeneously along the sample. Figure 9a shows that, in 5% (*w*/*v*) concentration, the pores are predominantly distributed in the range of 0–1 μm in diameter, with an average of 0.503 μm. When the concentration increased to 7.5% (*w*/*v*) (Figure 9b), the number of pores increased. These pores mainly had a diameter in the range of 0.5–1.5 μm, with a weighted average of 0.949 μm. On the other hand, the 10% concentration (*w*/*v*) (Figure 9c) had approximately the same number of pores as the 5% concentration (*w*/*v*). These pores were mainly in the same diameter range as those in the concentration of 7.5% (*w*/*v*), with a weighted average of 0.974 μm. Although this pore size showed an increment as concentration increased, considering all the pores shown in the micrographs, the weighted averages of diameter were 0.832, 1.188, and 1.030 μm for 5%, 7.5%, and 10% (*w*/*v*), respectively.

Further studies must be performed to evaluate biocompatibility and cytotoxicity when stents are implanted. These studies are essential to demonstrate the impact of the coating even without drugs included. Shomali et al. (2017) reported that zinc wires coated with PLLA caused an inflammatory response in neointimal tissue months after implantation [32]. This happened due to the release of toxic and acidic by-products of PLLA degradation.

### 3.3. Thin Film Characterization

**Figure 10:**
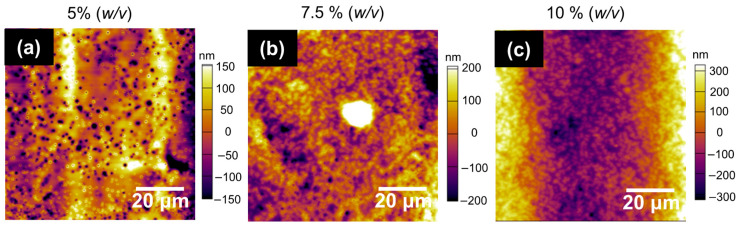
AFM images of PLA film at different concentrations: (**a**) 5% (*w*/*v*), (**b**) 7.5% (*w*/*v*), and (**c**) 10% (*w*/*v*).

The PLA film prepared with the process parameters selected at different concentrations, 5%, 7.5%, and 10% (*w*/*v*), was investigated using AFM (Figure 10). Figure 10a shows that a concentration of 5% (*w*/*v*) demonstrated irregularities on the surface; the black dots show porosity. The number of pores increased when the concentration increased to 7.5% (*w*/*v*) (Figure 10b) compared those at 5% (*w*/*v*). Figure 10c shows the images for a concentration of 10% (*w*/*v*), and the surface produced a more saturated image with black dots. The average roughness of each sample was 73.74, 111.75, and 194.13 nm for 5%, 7.5%, and 10% (*w*/*v*), respectively. These measurements were consistent with the pores observed for each image concentration.

Water contact angle measurements were obtained for coatings at different concentrations of 5%, 7.5%, and 10% (*w*/*v*) at the same conditions of process parameters selected (S_E_ = 500 mm min^−1^, S_W_ = 500 mm min^−1^ t = 15 s, C = 1). The samples were cleaned with acetone before the measurements were taken to ensure their cleanliness. Drops of deionized water (10 µL) were deposited along the sample.

Figure S1 shows no significant difference in the average water contact angle between the 5%, 7.5%, and 10% (*w*/*v*) concentrations. However, samples coated with 7.5% (*w*/*v*) had better hydrophilic properties than the other concentrations. According to Mani et al. (2009), cell adhesion was better on moderately wet surfaces because of the preferential adsorption of cell-adhesive proteins such as fibronectin and vitronectin onto these surfaces [60]. They showed that cell adhesion was excellent on surfaces with contact angles varying from 25° to 70°. Ren et al. (2022) also stated that it is essential for PLA to be hydrophilic for cell adhesion, and its nature tends to be hydrophobic [61]. However, in our study, the angles used showed that PLA is hydrophilic (73.53°, 73.28°, and 73.82° for 5%, 7.5%, and 10% (*w*/*v*), respectively). Further studies must evaluate the solution’s concentration to create a more hydrophilic surface, demonstrating the significant difference between the water contact angles. Additionally, cell adhesion must be confirmed depending on the hydrophilic surface level.

**Figure 11:**
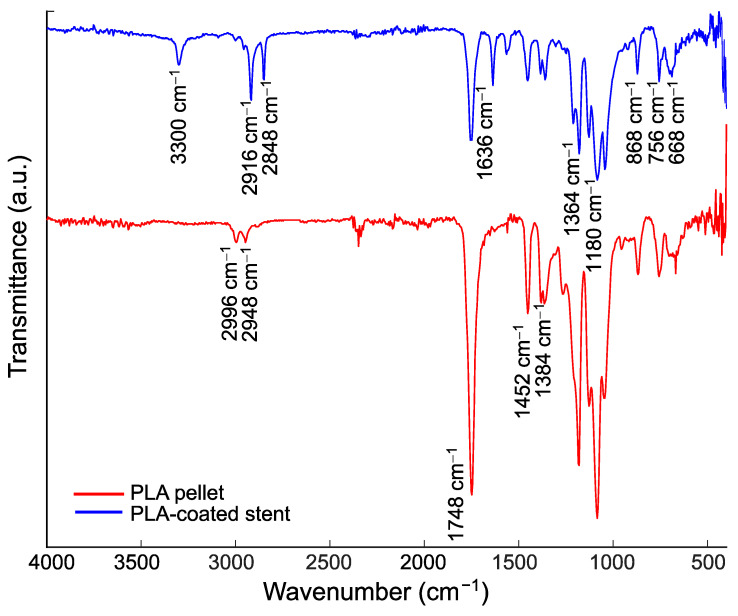
Fourier transform infrared (FTIR) spectra of PLA.

Figure 11 shows the FTIR spectra of the PLA pellet and PLA-coated stent to verify the presence of PLA film on the substrate. The comparison between the two spectra shows that they are very similar, albeit with some differences in peak intensities. The exhibited bands at 3300 cm^−1^ and 1636 cm^−1^ in the PLA pellet spectra correspond to O–H group vibrations and hydrated H_3_O^+^ [62,63,64]. The band detected at 1748 cm^−1^ was attributed to the stretching mode of carbonyl vibration C=O [65,66,67]. At 1452 and 1361 cm^−1^, there were bending frequencies for C–H asymmetric and symmetric stretching in the –CH_3_ methyl groups, respectively [67,68]. The peak at 1180 cm^−1^ represents the higher-intensity vibration of C–O [20] in the PLA-coated stent. The vibration band located at 1384 cm^−1^ was assigned to stretching vibrations in the carboxyl group [67]. Another band found at 868 cm^−1^ corresponds to C–C vibrations [66]. These peaks were more intense in the PLA pellet than in the PLA-coated stent. Conversely, two bands with higher intensities are observed in the PLA-coated stent in the 2996–2848 cm^−1^ range corresponding to –CH and –CH_3_ stretching frequencies [20,69].

**Figure 12:**
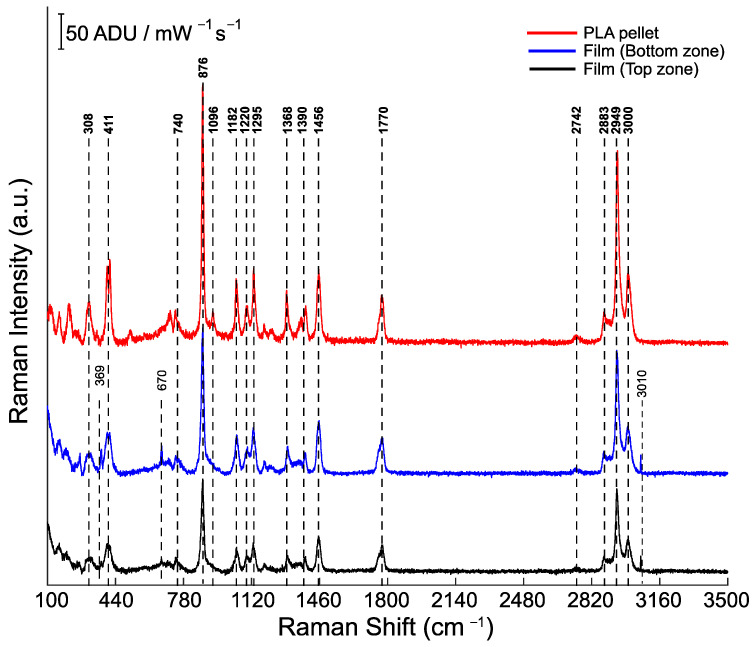
Raman spectra of PLA pellets and PLA film 7.5% (*w*/*v*).

Figure 12 compares the full Raman spectra of pure PLA and PLA film deposited on the SS sample in the 100–3500 cm^−1^ region. All spectra look very similar, and they show intensity changes. These changes mean that more material was accumulated in the bottom zone due to the sample’s position. Similar bands in all spectra include CH_3_ asymmetric and symmetric stretching vibrations assigned to 3000 cm^−1^ and 2949/2883 cm^−1^, respectively. At 1770 cm^−1^, there is CH stretching and strong C=O groups. CH_3_ symmetric deformation vibration is assigned to 1456 cm^−1^, and functional CH_3_ asymmetric groups which used to appear in the frequency range between 1384 and 1388 cm^−1^ shifted to 1390 cm^−1^ [70]. Bands at 1220 and 1182 cm^−1^ indicate a moderate C–O–C asymmetric group. The Raman spectra at frequency 1096 cm^−1^ have very strong C–O–C symmetric stretching. C–CH_3_ stretching was assigned to 1043 cm^−1^. C–COO vibration was assigned to 876 cm^−1^. Moderate C=O stretching groups are assigned to 740 cm^−1^, C–CO vibrational states to 411 cm^−1^, and C–O–C + C–CH_3_ vibration is given to 308 cm^−1^ [71].

On the PLA film spectra, the presence of a minimal amount of CHCl_3_ in the film after drying can be seen, shown by the presence of characteristic bands of CHCl_3_ at 369, 670, and 3010 cm^−1^. This residual solvent can be reduced by vacuum oven drying after the sample is coated. C–Cl vibrations are located at 369 and 670 cm^−1^, and the band at 3010 cm^−1^ is C–H vibration. Since there was no fluorescent background, it can be concluded that the number of residual solvent molecules interacting with the polymer was relatively small [72].

## 4. Conclusions

The 316 L SS pieces were laser cut, electropolished, coated, and characterized to obtain the best selection of parameters. The study of the PLA solutions demonstrated that critical entanglement concentration (C_e_) was obtained at around 7% (*w*/*v*) of PLA and a concentration of 7.5% (*w*/*v*) of PLA was used. From the process window, the uniformity was increased at entry and withdrawal speeds of 500 mm min^−1^, resulting in a more uniform coating of film along the tube, independent of immersion time. More material was adhered to the sample at high speeds (S_E_ = S_W_ = 500 mm min^−1^). The sample weighed 0.1190 mg when both speeds were at 100 mm min^−1^ and 5 s, and it increased to 0.1205 mg when both speeds were at 500 mm min^−1^ and 15 s. Surface roughness decreased at S_E_ = 500 mm min^−1^ and S_W_ = 100 mm min^−1^ with 15 s of immersion time, providing a value of 0.685 µm; it decreased by approximately 18% before coating.

To coat complex geometries such as stents, it was found that high speeds (500 mm min^−1^ for entry and withdrawal speed) and long immersion times (15 s) resulted in an average thickness of 7.862 µm. These conditions resulted in a uniform coating with a thickness between 4–10 µm and an adequate surface finish on the stent (~0.761 μm). Porosity was observed, which was caused by solvent evaporation. As concentration increases, thickness of porosity increases. At 7.5% (*w*/*v*), the weighted average of the pore diameter was the highest (1.188 µm). FTIR analysis confirmed the main functional groups in the PLA on the stent, demonstrating the polymer film’s presence compared to the pellet’s functional groups. Raman confirmed that more material was in the bottom zone when the process had just one cycle. For future studies, optimizing the position of the sample to minimize the thickness differences throughout the sample due to gravity is crucial.

## Figures and Tables

**Table 2:** Dip coating process parameters for process window.

Parameters	Values (Low, High)
Entry Speed, S_E_ [mm min^−1^]	100, 500
Withdrawal Speed, S_W_ [mm min^−1^]	100, 500
Immersion Time, t [s]	5, 10, 15
Cycles, C	1

## Data Availability

The data presented in this study are available on request from the corresponding author upon reasonable request.

## Supplementary Materials

The following supporting information can be downloaded at: https://www.mdpi.com/article/10.3390/polym16020284/s1, Table S1: Surface tension results of PLA solutions with different concentrations; Figure S1: Average water contact angle for the three concentrations.
