# Supplementary material for: A Study of PLA Thin Film on SS 316L Coronary Stents Using a Dip Coating Technique
Source: Polymers (Basel). 2024 Jan 19;16(2):284. doi: 10.3390/polym16020284 (PMC10818791; doi:10.3390/polym16020284)
Supplement: Supplementary file 1 [file polymers-16-00284-s001.zip › polymers-2784798-supplementary.pdf]

Supplementary file

Table S1. Surface tension results of PLA solutions with different concentrations.

PLA concentration (% (*w*/*v*))	Surface tension (mN m⁻¹)
0.5	25.36 (± 0.14)
1.0	25.33 (± 0.28)
3.0	25.10 (± 0.27)
5.0	25.23 (± 0.08)
7.5	24.56 (± 0.46)
10.0	25.27 (± 0.58)
12.5	24.68 (± 0.08)
15.0	26.17 (± 0.17)

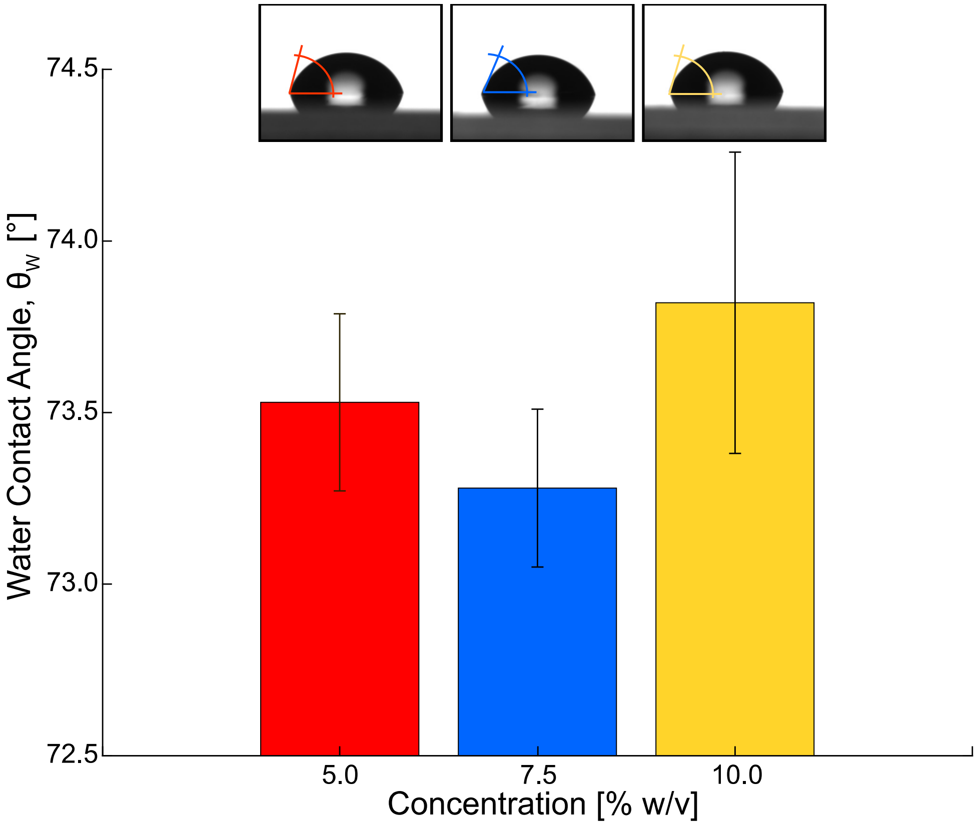

Figure S1. Average water contact angle for the three concentrations.
